# The Major Constituent of Green Tea, Epigallocatechin-3-Gallate (EGCG), Inhibits the Growth of HPV18-Infected Keratinocytes by Stimulating Proteasomal Turnover of the E6 and E7 Oncoproteins

**DOI:** 10.3390/pathogens10040459

**Published:** 2021-04-11

**Authors:** Jason K. W. Yap, Sean T. Kehoe, Ciaran B. J. Woodman, Christopher W. Dawson

**Affiliations:** 1 Institute for Cancer & Genomic Sciences, College of Medicine & Dentistry, University of Birmingham, Birmingham B15 2TT, UK; s.t.kehoe@bham.ac.uk (S.T.K.); c.b.woodman@bham.ac.uk (C.B.J.W.); 2 Pan-Birmingham Gynaecological Cancer Centre, City Hospital, Dudley Road, Birmingham B18 7QH, UK; 3 Warwick Medical School, Gibbet Hill Campus, University of Warwick, Coventry CV4 7AL, UK; C.Dawson.3@warwick.ac.uk

**Keywords:** human papilloma virus, vulval intraepithelial neoplasia, epigallocatechin-3-gallate

## Abstract

Epigallocatechin-3-gallate (EGCG), the primary bioactive polyphenol in green tea, has been shown to inhibit the growth of human papilloma virus (HPV)-transformed keratinocytes. Here, we set out to examine the consequences of EGCG treatment on the growth of HPV18-immortalised foreskin keratinocytes (HFK-HPV18) and an authentic HPV18-positive vulvar intraepithelial neoplasia (VIN) clone, focusing on its ability to influence cell proliferation and differentiation and to impact on viral oncogene expression and virus replication. EGCG treatment was associated with degradation of the E6 and E7 oncoproteins and an upregulation of their associated tumour suppressor genes; consequently, keratinocyte proliferation was inhibited in both monolayer and organotypic raft culture. While EGCG exerted a profound effect on cell proliferation, it had little impact on keratinocyte differentiation. Expression of the late viral protein E4 was suppressed in the presence of EGCG, suggesting that EGCG was able to block productive viral replication in differentiating keratinocytes. Although EGCG did not alter the levels of E6 and E7 mRNA, it enhanced the turnover of the E6 and E7 proteins. The addition of MG132, a proteasome inhibitor, to EGCG-treated keratinocytes led to the accumulation of the E6/E7 proteins, showing that EGCG acts as an anti-viral, targeting the E6 and E7 proteins for proteasome-mediated degradation.

## 1. Introduction

Persistent infection with high-risk human papilloma virus (HR-HPV) strains is causally linked to the development of usual type vulvar intraepithelial neoplasia (uVIN) [1]. In most cases, the virus is maintained as extrachromosomal episomes in infected basal keratinocytes, undergoing genome amplification and productive viral replication as cells mature and differentiate [2]. uVIN primarily affects young women, with a peak age incidence of 30–49 years; however, the incidence of uVIN has increased by more than three-fold in recent years [3,4]. Although the malignant potential of uVIN is significantly lower than that of cervical intraepithelial neoplasia (CIN) [5], it often causes debilitating symptoms such as pruritus, pain and sexual dysfunction. Moreover, at least 50% of women were found to have multiple uVIN lesions (multifocal disease), and one in three women also found to have synchronous or asynchronous multicentric intraepithelial neoplasia of the cervix, vagina and anus [6]. Currently, surgical excision remains the mainstay of treatment for women with uVIN, but it does not offer a cure. More than a third of these women will have recurrences, and surgery may itself result in additional physical and psychosexual problems [7]. Novel alternatives to surgery have been investigated, for example, the use of the topical anti-viral Cidoforvir or the immune modular Imiquimod, but both have yielded variable results [8,9,10]. Towards this end, we have recently completed a Phase II randomised control trial (EPIVIN) evaluating the use of a novel topical therapeutic agent, Sinecathecin, in the treatment of women with uVIN and found that the treatment is safe and shows promise in inducing clinical resolution of uVIN and symptom improvement [11].

EGCG has been formulated into a topical ointment, namely, Sinecatechins, and licensed for the treatment of genital warts, a proliferative disorder induced by low-risk HPV subtypes, notably, HPV6 and HPV11 [12]. Sinecathecin ointment contains epigallocathecin-3-gallate (EGCG), a major bioactive polyphenol of green tea which has been shown to possess multiple anti-carcinogenic effects in cell culture and animal models of cancer and, more importantly, shown to be safe and effective in treating proliferative disorders associated with low-risk HPV strains [12]. Its biological activities, which are broad-ranging, have been investigated in many human diseases, including cancer [13]. However, to date, the underlying mechanism of action of EGCG in these low-risk HPV-driven epithelial disorders remains unknown, and its use for the treatment of genital warts was not founded on experimental evidence. Nevertheless, it has also been shown to target a host of potential oncogenic viruses such as Epstein–Barr virus, (EBV), hepatitis B virus (HBV) and human immunodeficiency virus (HIV) by inhibiting mechanisms of viral replication, gene expression or viral assembly [14].

Several studies have shown that EGCG reduces transcription of the HR-HPV-encoded E6 and E7 proteins in cervical cancer-derived cell lines [15,16,17], a phenomenon associated with upregulation of the E6 and E7 targets p53, pRb and p21^WAF1^. Unfortunately, it is still unclear whether upregulation of these tumour-suppressor genes (TSGs) is a consequence of E6 and E7 downregulation or occurs through direct stimulation of signalling pathways that affect their expression. EGCG has been shown to stimulate the expression of these TSGs by modulating the expression of key epigenetic modulators such as histone deacetylase (HDAC), DNA methyltransferases (DMNTs) and the polycomb group (PcG) of proteins [18,19,20,21]. Also, a small number of studies have examined the effects of EGCG on the growth and differentiation of normal epidermal keratinocytes and carcinoma cell lines in vitro and in vivo. Collectively, these studies show that EGCG promotes differentiation by inducing cell-cycle withdrawal and stimulating the expression of a range of differentiation-associated genes [18,22,23,24]. This effect might indirectly affect the expression of viral oncogenes.

To our knowledge, the effect of EGCG on the HPV life cycle has not been explored in great detail. Based on current evidence, we hypothesised that EGCG might act in several ways. It may directly disrupt episomal maintenance by targeting E6, E7 and the genome maintenance proteins, or act indirectly, by modulating keratinocyte differentiation in such a way that infected cells are no longer able to maintain viral episomes, forcing the virus to undergo abortive or productive infection. Using primary human foreskin keratinocytes stably infected with episomal forms of HPV18 and an authentic HPV18-positive cell line isolated from a primary vulvar intraepithelial neoplasia (VIN), we examined the effects of EGCG treatment on keratinocyte proliferation and differentiation in both monolayer and organotypic raft culture, focusing specifically on the expression of the key viral oncoproteins E6 and E7. Our primary objective was to elucidate the underlying mechanism(s) of action of EGCG in HR-HPV and, in doing so, establish whether long-term treatment with a topical EGCG ointment is effective against uVIN.

We took the initiative to isolate our own VIN cell line for two reasons. Firstly, the uVIN-derived cell lines previously described [25,26] were not available and, secondly, both cell lines contained integrated forms of HPV16 precluding an assessment of the effects of EGCG on the HPV life cycle.

## 2. Results

### 2.1. The Cell Models: HFK-HPV18 and VIN cl.11

**Figure 1:**
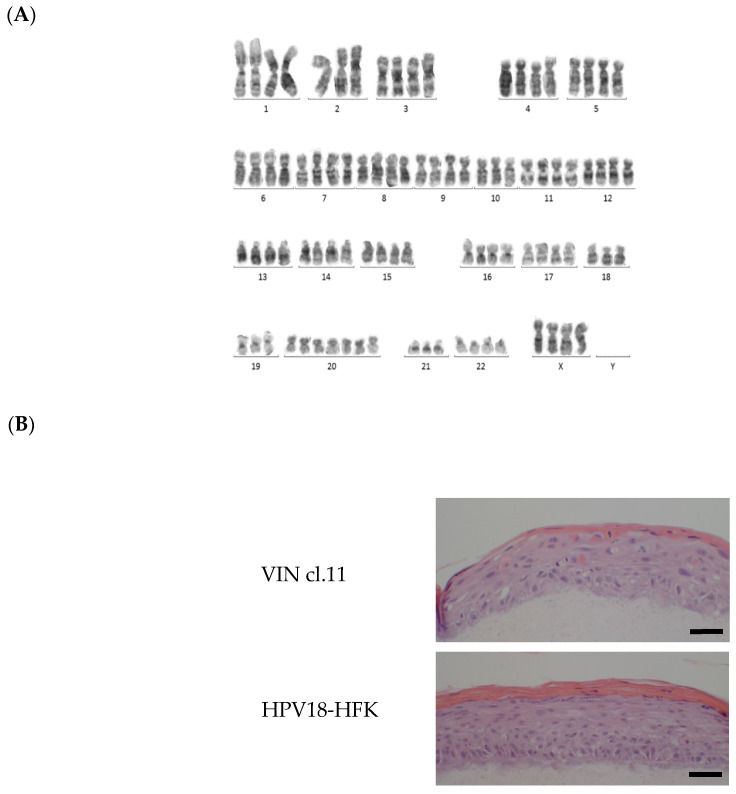
Characterisation of human papilloma virus (HPV)18-positive usual vulvar intraepithelial neoplasia (uVIN)-derived clone—VIN cl.11. (**A**) A representative karyotype from one of three major clones identified in primary cultures of the VIN cl.11. Chromosome alignment of G-banded chromosomes taken from one major clone found in early-passage cultures of VIN cl.11. This analysis confirmed the near-tetraploid nature of chromosomes and the absence of a Y chromosome. (**B**) Hematoxylin and eosin (H&E)-stained sections from organotypic raft cultures grown at the air–liquid interface for 14 days. Both VIN cl. 11 and HFK-HPV18 rafts displayed evidence of parakeratosis, but VIN cl.11 appeared less organized, with areas of immature and large nucleated cells extending to the granular layer, a feature suggestive of dysplastic changes that resembles uVIN. Scale = 10 μm.

The cell models employed in this study included human foreskin keratinocytes (HFK)-HPV18, an immortalised foreskin keratinocyte cell line containing episomal forms of HPV18 [27], and VIN clone 11, a clone of keratinocytes generated by single-cell cloning of a primary VIN biopsy culture [28]. Chromosome analysis, performed on early-passage cultures of VIN cl.11, revealed a female chromosome complement and an abnormal karyotype that were near tetraploid, consistent with neoplasia (Figure 1A; Supplementary Figure S1). The HPV status of the VIN-derived cell line (VIN cl.11) was determined using a Luminex-based PCR assay [29], and the cell line was found to harbour HPV18. As with normal vulval keratinocytes and HFK-HPV18, VIN cl.11 also formed undifferentiated colonies in monolayer culture (Supplementary Figure S2) and exhibited features of dysplasia in organotypic raft culture [30].

### 2.2. EGCG Inhibits Proliferation and Induces Apoptosis of HFK-HPV18 and VIN cl.11 Keratinocytes Grown in Monolayer Culture

**Figure 2:**
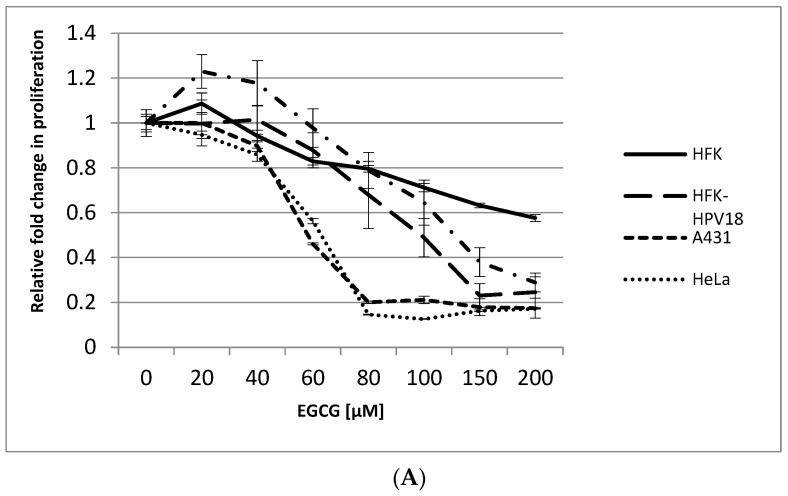

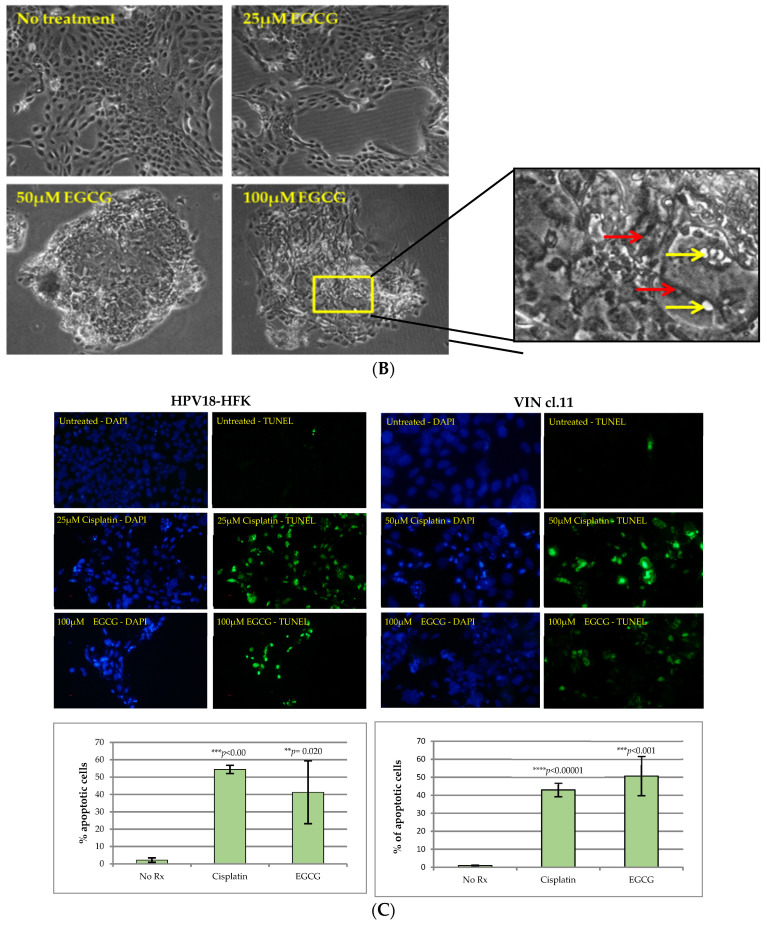
Epigallocathecin-3-gallate (EGCG) inhibits cell proliferation and induces apoptosis in HFK-HPV18 and VIN cl.11. (**A**). Normal human foreskin keratinocytes (HFK), HPV18-positive keratinocytes (HFK-HPV18, VIN cl.11) and cancer cell lines (A431, HeLa) were treated with increasing concentrations of EGCG, and proliferation was measured 72 h later using the BrdU ELISA assay kit (Roche). The fold change in proliferation of EGCG-treated cells was measured against untreated cells (control). Cell proliferation decreased as the concentration of EGCG increased. Data shown are the average of three independent experiments. (**B**). HFK-HPV18 cells were cultured in in the presence of 25, 50 and 100 μM EGCG for 72 h, and cell morphology was captured by phase microscopy Magnification ×200. Inset: high-power close-up of intracellular vacuoles in cells treated with 100 μM EGCG. EGCG treated cells assumed a spindle-like appearance (red arrows) with intracellular vacuole (yellow arrows) at 100µM. Magnification ×400. (**C**). HFK-HPV18 and VIN cl.11 cells were cultured in the presence of 100 μM EGCG for 72 h or 25 μM cisplatin for 24 h; the latter was used as a positive control to induce apoptosis. TUNEL assay was used to label apoptotic cell (Green), and cell nuclei were counterstained with DAPI (blue). No Rx = non treated. Magnification ×200. TUNEL-positive cells were expressed as a percentage of total cell nuclei. Unpaired Student *t*-test was used to determine the level of significance for the difference in the proportion of apoptotic cells in drug-treated and untreated cells (n = 3) (** *p*-value <0.05, *** *p*-value 0.001, **** *p*-value 0.0001).

In monolayer culture, EGCG induced a dose-dependent decrease in cell proliferation in all cell lines tested (Figure 2A). HeLa and A431 cells were included as positive controls, given their known responsiveness to EGCG treatment [15,20]. The IC_50_, a concentration of drug at which 50% of cell proliferation is inhibited, was 60 μM for HeLa and A431 and 100 μM and 150 μM for HFK-HPV18 and VIN cl.11, respectively. Interestingly, the highly transformed cervical (HeLa) and vulvar (A431) cancer cell lines were more sensitive to EGCG treatment than the HPV18-positive non-malignant keratinocyte cell lines. Also included in this study was HFK, the normal isogenic counterpart of HFK-HPV18; unlike HFK-HPV18, the proliferation of HFK was only marginally affected by EGCG treatment. Phase microscopy revealed that EGCG-treated cells developed cytoplasmic vacuoles, assumed a spindle-like appearance and showed signs of nuclear degeneration, changes that were most apparent in cells treated with 100 μM EGCG (Figure 2B). Examination of EGCG-treated cells for evidence of apoptosis was performed using the TUNEL assay. Staining revealed that EGCG treatment led to a robust induction of apoptosis, with over 40% of HFK-HPV18 and VIN cl.11 cells undergoing apoptosis after 72 h of EGCG treatment. Cisplatin, a genotoxic agent known to induce apoptosis, was included as a positive control, inducing apoptosis in approximately 40–50% of cells after a 24 h treatment (Figure 2C).

### 2.3. EGCG Inhibits HFK-HPV18 and VIN cl.11 Proliferation in Organotypic Raft Culture

**Figure 3:**
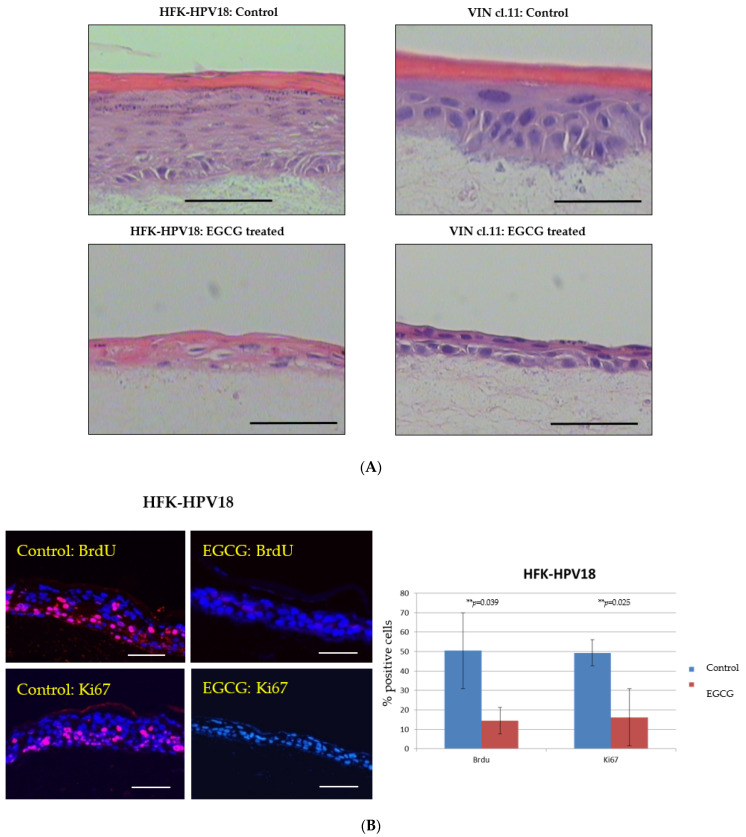

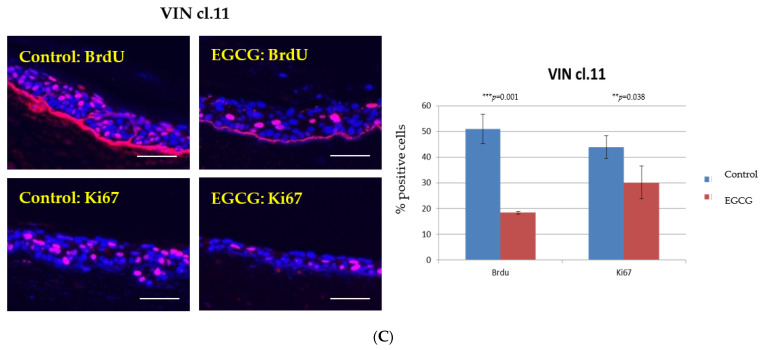
EGCG inhibits the proliferation of HFK-HPV18 and VIN cl.11 in organotypic raft culture. (**A**) EGCG inhibits the proliferation of HFK-HPV18 (top) and VIN cl.11 (bottom) in organotypic raft culture. Raft cultures were allowed to stratify for 7 days before 100 μM EGCG was added to the growth media for an additional 7 days prior to fixation and processing for histology and histocytochemical staining. H&E-stained sections of formalin-fixed paraffin embedded (FFPE) samples showing the overall morphology of HFK-HPV18 and VIN cl.11 cells grown in organotypic raft culture. Rafts cultures treated with 100 μM EGCG were significantly thinner compared to control (untreated) raft cultures. Scale = 5 μm. (**B**) Immunofluorescence staining for anti-BrdU label or Ki67 (Pink) and with DAPI counterstaining (Blue) to identify cell nuclei in (**B**) HFK-HPV18 and (**C**) VIN cl.11 raft sections (Right panels). Scale = 5 μm. Summary of the results obtained for cells incorporating BrdU label or staining positive for Ki67 in control and EGCG-treated raft cultures. The total number of cell nuclei (DAPI-stained) and those nuclei labelled with BrdU or Ki67 were counted manually. Results are presented as the proportion of cells stained positive for the appropriate proliferative markers. ** *p* < 0.05, *** *p* < 0.001, two-tailed Student unpaired *t*-test indicates that the difference in BrdU or Ki67 expression is significant when compared to control (n = 3).

Next, we examined the effect of EGCG on cells cultured in organotypic raft culture. HFK-HPV18 and VIN cl.11 cells were allowed to stratify for 7 days at the air–liquid interface and then exposed to 100 μM EGCG for a further 7 days prior to fixation and processing. Figure 3A shows that EGCG-treated raft cultures formed significantly thinner epithelia compared to untreated rafts. Immunofluorescence staining of raft sections for BrdU label or Ki67 revealed a significant reduction in nuclear staining in response to EGCG treatment, indicating that DNA synthesis and cell proliferation were inhibited by greater than 50% in both HFK-HPV18 and VIN cl.11 cells in raft culture (Figure 3B,C).

### 2.4. EGCG Downregulates the Expression of the E6 and E7 Viral Oncogenes by Enhancing Their Turnover

**Figure 4:**
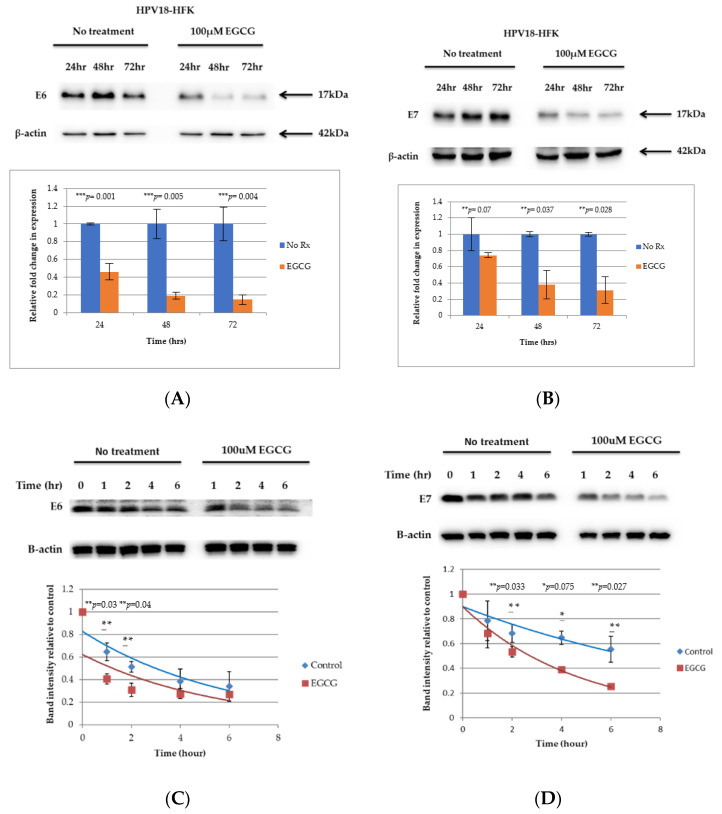
EGCG downregulates E6 and E7 in HFK-HPV18 keratinocytes by enhancing their turnover. (**A**,**B**) HFK-HPV18 cells were treated with 100 μM EGCG for 24, 48 and 72 h, and 30 μg of total protein lysate was resolved by SDS-PAGE. The levels of (**A**) E6 and (**B**) E7 were then determined by Western immunoblotting analysis. Densitometric analysis of representative Western blots for E6 and E7 were normalised against β-actin and are shown in graphical form. Fold change in protein expression was compared to that of untreated cells (control). Unpaired Student *t*-test confirmed that the difference in protein expression was significant between control and EGCG treated cells (** *p*-values 0.01–0.009; *** *p*-values 0.001–0.005) (n = 3). HFK-HPV18 cells were treated with and without 100 μM EGCG in the presence of 100 μg/mL cycloheximide (CHX) to inhibit protein synthesis. Cells were harvested at 0, 1, 2, 4 and 6 h post CHX treatment, 30 μg of total protein lysate was resolved by SDS-PAGE and expression of either (**C**) HPV18 E6 or (**D**) HPV18 E7 and β-actin was determined by Western blotting analysis. Densitometric analysis of the Western blots. E6 and E7 densitometry values were normalised against β-actin. The fold change in E6 and E7 expression was compared against that of untreated cells (control). Unpaired Student *t*-test indicates that the difference in band intensity was significant between control and EGCG treated cells (** *p*-values of <0.05, * *p*-value of <0.1) (n = 3).

Having established that EGCG inhibits proliferation and promotes apoptosis in both HFK-HPV18 and VIN cl.11 cells, we next examined the effect of EGCG on the expression of the E6 and E7 proteins, given their critical role in HR-HPV-driven cell proliferation. Western blotting analysis showed that the levels of E6 protein were reduced by more than 50% following a 24 h treatment with EGCG and continued to decrease further after 48 and 72 h of treatment (Figure 4A—left panel). Similarly, the E7 protein started to decline 24 h after EGCG treatment, with levels remaining lower than that of the untreated control after 48 and 72 h of treatment (Figure 4A—right panel). To further strengthen the notion that EGCG stimulates the turnover and degradation of the E6 and E7 proteins, the half-life E6 and E7 was measured in the absence and presence of EGCG. HFK-HPV18 keratinocytes were treated with 100 μg/mL cycloheximide (CHX) in the presence or absence of 100 μM EGCG over a period of 1 to 6 h, and the levels of E6 and E7 were measured by Western blotting. A representative experiment, Figure 4C,D, shows that the rate of E6 and E7 degradation was increased significantly within the first hour following EGCG treatment. The half-life of E6 and E7 was approximately 3 h, but this was significantly reduced to just under an hour following EGCG treatment. The rate of protein degradation was at its peak in the first 1.5 h after EGCG treatment and, thereafter, appeared to stabilize, with the rate of protein degradation similar to that of untreated controls.

### 2.5. EGCG Downregulates the Viral Oncogenes E6 and E7, Leading to Re-Expression of Their Target TSGs

**Figure 5:**
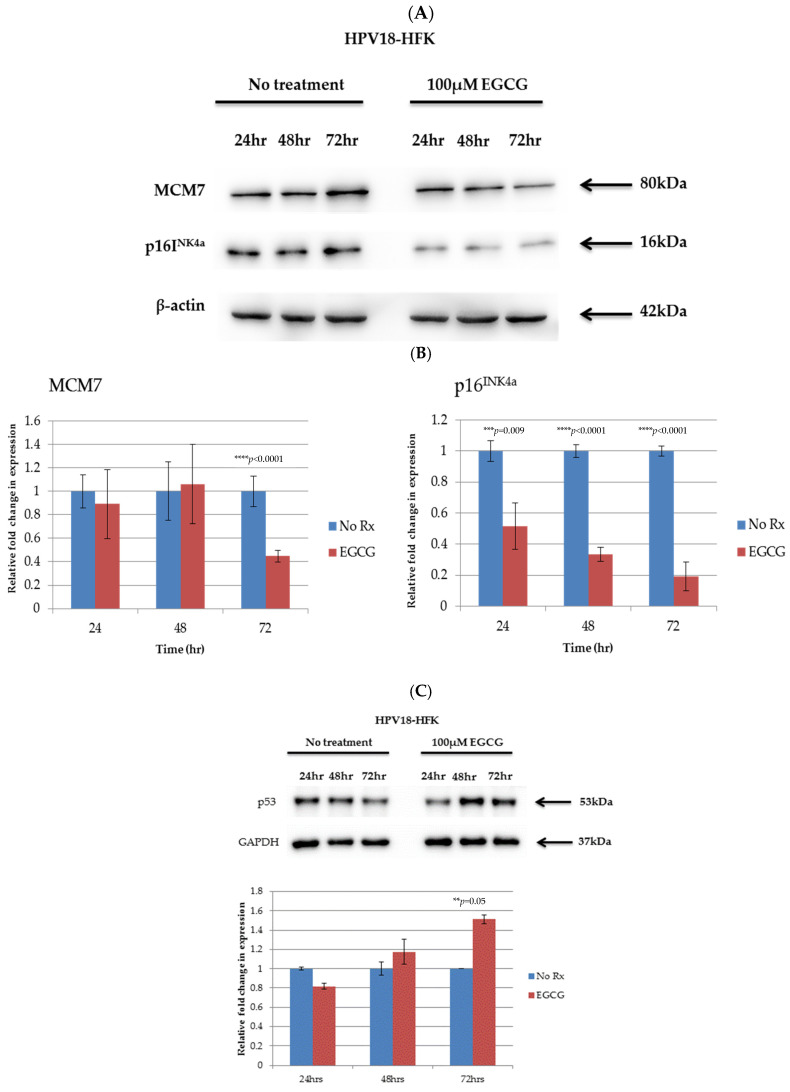
Downregulation of HPV-18 E6 and E7 and altered expression of E6- and E7-associated targets (MCM7, p16^INK4a^, DNMT1/3 and p53) in HFK-HPV18 keratinocytes. HFK-HPV18 cells were treated with 100 μM EGCG for 24, 48 and 72 h, and 30 μg of total protein lysate was resolved by SDS-PAGE. (**A**) The levels of MCM7 and p16^INK4a^ proteins were then determined by Western immunoblotting analysis. (**B**) Densitometric analysis of representative Western blots for E6, E7, MCM7 and p16^INK4a^ were normalised against β-actin and shown in graphical form. Fold change in protein expression was compared to that of untreated cells (control). ** *p* < 0.05. Unpaired Student *t*-test was used to determine if the difference in protein expression was significant when compared to control (n = 3). No Rx = No treatment (**C**) HFK-HPV18 cells were treated with 100 μM EGCG for 24, 48 and 72 h, and the levels of p53 were determined by Western blotting analysis. Densitometry analysis of the blots (lower panel). p53 densitometry values were normalised against GAPDH. The fold change in p53 expression was compared against untreated cells (No Rx). Student unpaired *t*-test indicated that the difference in p53 expression was significant between control and EGCG treated cells (** *p*-value 0.05; *** *p*-value 0.001–0.005; **** *p*-value 0.0001) (n = 3).

Interestingly, downregulation of the E6 and E7 proteins was associated with an upregulation of the E6 and E7 target genes MCM7 and p16INK4a (Figure 5A,B). We also compared the expression of p53 in control and EGCG-treated HFK-HPV18, given that EGCG has been shown to alter the expression of this gene [20]. We found that EGCG treatment stimulated the expression of p53 in HFK-HPV18, with levels of p53 protein increasing 48 h and 72 h post treatment (Figure 5C). Our results suggest that the changes in expression of TSGs observed in EGCG-treated HFK-HPV18 cells are a consequence of the downregulation E6 and E7 and not due to direct stimulation by EGCG. Collectively, our findings indicate that EGCG downregulates the expression of HPV oncogenes, which allows the re-expression of their target TSGs, resulting in growth inhibition and apoptosis.

### 2.6. EGCG Promotes E6 and E7 Degradation through the Proteasome

**Figure 6:**
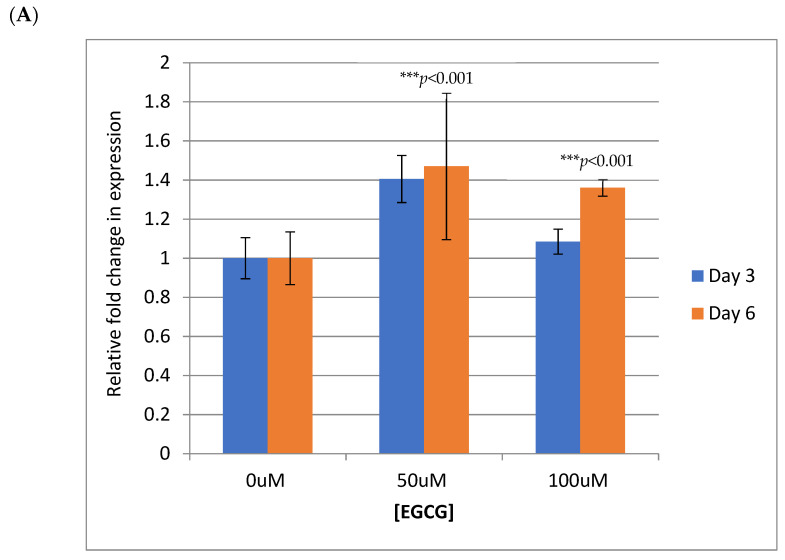

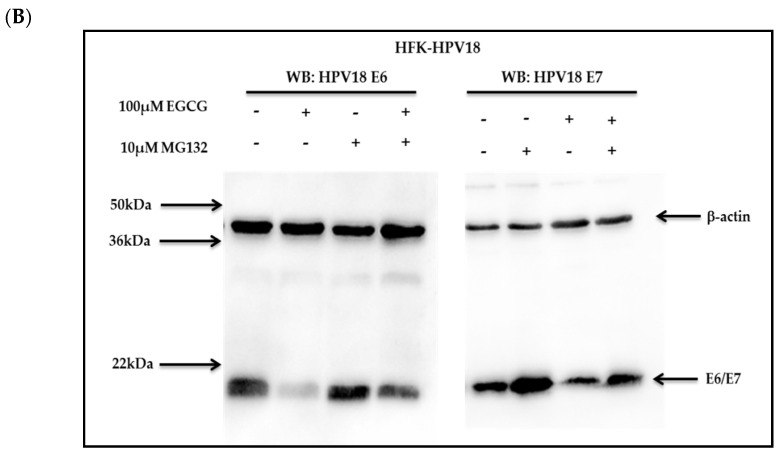
EGCG does not affect E6/E7 transcription but promotes degradation of the E6 and E7 proteins by enhancing their turnover through the proteasome. (**A**) HFK-HPV18 cells were treated with 50 μM or 100 μM EGCG for 3 and 6 days. RNA was isolated, andqPCR performed to quantify the relative fold change in E6/E7 transcripts pre- and postEGCG treatment. Expression levels were normalised to levels of endogenous β2-microglobulin in the samples. Data were analysed using the relative 2ΔΔCT method using 7500 SDS software (n = 3). *** denotes a *p*-value of <0.001 by Student unpaired *t*-test. (**B**). HFK-HPV18 cells were treated with 10 μM MG132 for 6 h, 100 μM EGCG for 72 h or 100 μM EGCG for 72 h followed by 10 μM MG132 for 6 h. Cells were lysed in RIPA buffer, and 30 μg of total protein lysate was resolved by SDS-PAGE. The levels of HPV18 E6, E7 and β-actin were determined by Western immunoblotting analysis (n = 3).

Next, we examined the mechanism by which EGCG downregulates the expression of E6 and E7. To explore the possibility that the reduction in HPV18 E6 and E7 proteins occurred as a result of decreased transcription, the levels of E6 and E7 mRNA were examined in HFK-HPV18 by q-PCR. Our analysis revealed that the levels of E6 and E7 mRNA transcripts were not affected by EGCG treatment (Figure 6A). The findings here suggest that the downregulation of E6 and E7 proteins following EGCG treatment does not occur through effects on transcription of E6/E7 mRNA.

To establish whether EGCG enhances E6 and E7 degradation through the ubiquitin–proteasome pathway, MG132, a commonly used proteasome inhibitor, was used to block proteasome activity in untreated and EGCG-treated HFK-HPV18 keratinocytes. Cells were treated with 100 μM EGCG for 72 h to induce maximum E6 and E7 downregulation before the addition of 10 μM MG132 for the final 6 h; HFK-HPV18 keratinocytes were also treated with MG132 alone. Consistent with previous result, western blotting analysis confirmed that the levels of E6 and E7 protein were both downregulated following a 72 h treatment with of EGCG; however, MG132 treatment led to the accumulation of E6 and E7 protein, albeit to lower levels than those observed in untreated cell (Figure 5B). This suggests that both proteins are degraded through the ubiquitin–proteasome pathway and indicates that EGCG stimulates E6 and E7 protein turnover by enhancing their degradation through this pathway.

### 2.7. Inhibition of E6 and E7 Expression by EGCG Impairs the Lytic Replication of HPV18

**Figure 7:**
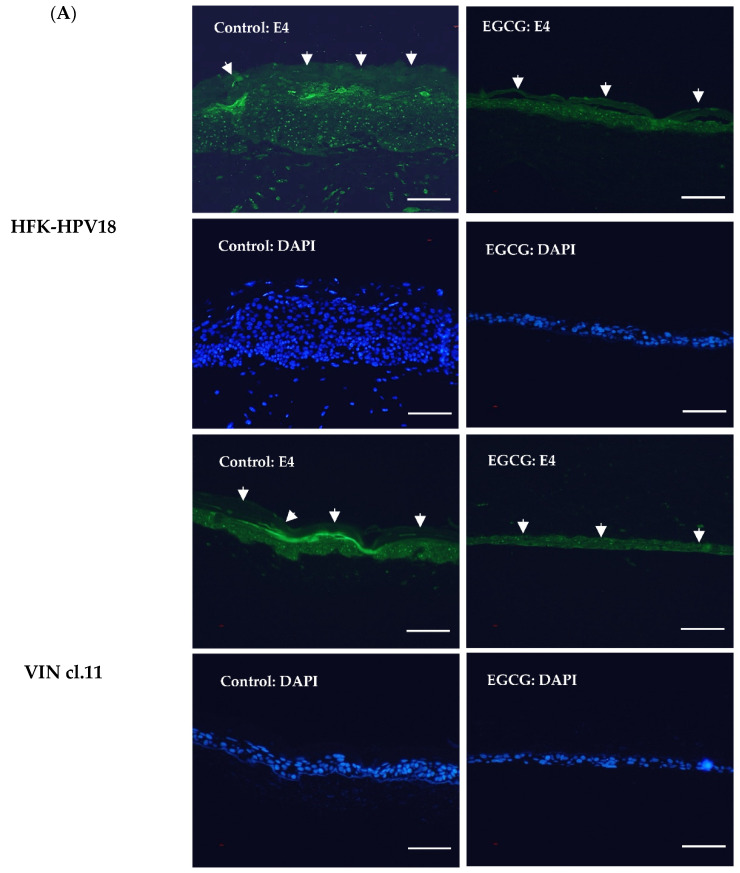

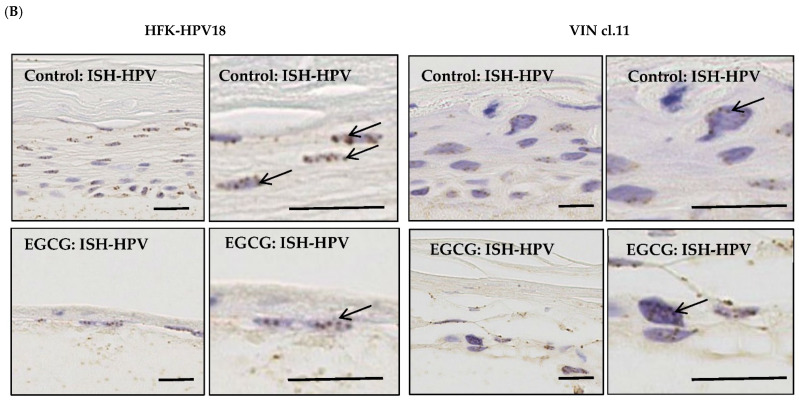
EGCG treatment inhibits the expression of the replication-associated protein E4 but does not influence HPV genome copy number in raft cultures. (**A**) FFPE sections of control and EGCG-treated HFK-HPV18 and VIN cl.11 rafts were subjected to immunocytochemical staining with an antiserum specific for HPV18 E4 (Green) and counterstained with DAPI (Blue) to label cell nuclei. Positive staining for the E4 protein in suprabasal keratinocyte layers of rafts generated from HFK-HPV18 and VIN cl.11 keratinocytes, but a lack of staining in rafts treated with EGCG (n = 3). Thick white arrows denote the outermost differentiated layers in HFK-HPV18 and VIN cl.11. Scale = 5 μm. (**B**) The same sections were subjected to in situ hybridisation (ISH) using an HPV-specific DNA probe. Specific binding of the HPV-specific DNA probe (denoted by black arrows) was observed in the nuclei of both HPV18-infected cell lines. EGCG treatment did not alter the intensity of staining of the HPV DNA probe (n = 3). Scale = 5 μm.

To establish if the HPV18 life cycle is also affected by the downregulation of the E6 and E7 proteins, we investigated the impact of EGCG treatment on HPV lytic replication in organotypic raft culture. Previous studies have confirmed suprabasal expression of the E4 protein in HFK-HPV18 keratinocytes induced to differentiate in the organotypic raft culture system [27]. E4 is a late protein whose expression is induced during the lytic phase of the virus life cycle [31]. In keeping with previous studies, immunofluorescence (IF) staining of HFK-HPV18 and VIN cl.11 raft sections revealed suprabasal expression of E4 in a proportion of differentiating cells in both HFK-HPV18 and VIN cl.11 rafts. In contrast, EGCG-treated rafts lacked expression of the E4 protein in the suprabasal differentiating cell layers, implying that entry of the virus into the lytic phase was disrupted in response to EGCG treatment (Figure 7A). In situ hybridization (ISH) on raft cultures generated from HFK-HPV18 and VIN cl.11 confirmed the presence of HPV18 DNA in the nuclei of control and EGCG-treated rafts; however, due to the poor integrity of EGCG raft cultures following ISH processing, we could not determine the impact of EGCG treatment on HPV18 DNA load or copy number (Figure 7B).

### 2.8. EGCG Treatment Does Not Grossly Affect Keratinocyte Differentiation

**Figure 8:**
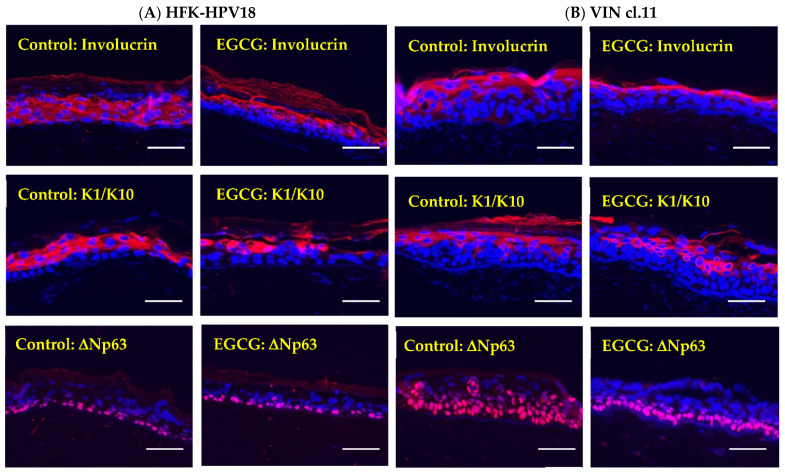
EGCG treatment is not associated with significant changes in the expression of keratinocyte differentiation proteins in HFK-HPV18 and VIN cl.11 rafts. FFPE sections of (**A**) HFK-HPV18 and (**B**) VIN cl.11 rafts were stained with antisera specific for involucrin, K1/10 and ΔNp63 (Pink) and counterstained with DAPI (Blue) to label cell nuclei. Similar patterns of expression of involucrin and K1/10 were observed in control and EGCG-treated rafts. However, while there was no change in the expression and localisation of ΔNp63 in control and EGCG-treated HFK-HPV18 rafts, ΔNp63 staining became “re-polarised” to the basal layer in EGCG-treated VIN cl.11 raft cultures compared to control untreated rafts (n = 3). Scale = 5 μm.

As lytic replication of HPV is intimately linked to keratinocyte differentiation, it is likely that disruption of this process can indirectly affect viral replication [32]. To determine whether the lack of viral replication was due to an effect of EGCG treatment on keratinocyte differentiation, control and EGCG-treated raft sections were stained with antibodies specific for several differentiation-associated proteins. IF staining (Figure 8) revealed for involucrin and K1/10 showed that similar levels of expression were observed in the suprabasal differentiating layers of control and EGCG-treated HFK-HPV18 and VIN cl.11 rafts. Objectively, there were no gross changes in the expression of these differentiation markers in EGCG-treated and untreated rafts, indicating that keratinocyte differentiation was not profoundly affected by EGCG treatment. In contrast, ΔNp63, a protein that identifies basal cells, was confined to the basal cell layer in HFK-HPV18 rafts (Figure 8) but expressed throughout most cell layers in VIN cl.11 rafts. While the levels and distribution of ΔNp63 were not altered in HFK-HPV18 rafts in response to EGCG treatment, its distribution was altered significantly in VIN cl.11 rafts, where ΔNp63 positive cells were confined to the basal layer following EGCG treatment. Collectively, our results suggest that EGCG treatment does not influence terminal differentiation but does impact on the expansion of the basal cells layer in VIN cl.11.

## 3. Discussion

While a substantial body of evidence has shown that green tea polyphenols, particularly EGCG, inhibit the growth and promote apoptosis of HR-HPV-positive cervical cancer cell lines [15,16,33], the underlying mechanism of action(s) still remains unclear. Of the studies that have evaluated the effects of EGCG treatment on cervical cancer cell lines in vitro, only two have examined the effects of EGCG treatment on the expression of the HPV-encoded E6 and E7 oncogenes. To this end, we set out to examine the effects of EGCG treatment on the growth and differentiation of premalignant HPV18-infected keratinocytes with a view to assessing its anti-viral properties.

In our study, we show that EGCG does indeed act as an anti-viral, targeting the E6 and E7 oncoproteins for proteasome-mediated degradation [34]. A consequence of this downregulation is re-expression of the E6 and E7 targets p53, p21^WAF1^ and pRb and a marked decrease in cell proliferation. EGCG has previously been shown to induce apoptosis in a range of cancer cell lines by directly stimulating the expression of a number of TSGs, particularly p53. Consistent with other studies, we found that the accumulation of p53 protein was associated with upregulation of its downstream target gene p21^WAF1^ (data not shown), which may be linked to the corresponding inhibition in cell proliferation [35].

Although EGCG has been shown to stimulate the expression of TSGs, we believe that the upregulation of p53 and pRb observed in EGCG-treated HPV18-positive keratinocytes is a consequence of E6 and E7 downregulation and not a direct effect of EGCG. This is based on our observations that EGCG failed to induce p53 and pRb and had only marginal effects on the proliferation of normal, non-HPV18-infected HFK.

Contrary to a previously published report [15], we observed little if any effect of EGCG treatment on the expression of E6 and E7 mRNAs, whereas immunoblotting confirmed an effect of EGCG on the levels of the E6 and E7 proteins. Previous studies have shown that the E6 and E7 oncoproteins are degraded through the proteasome [34]. We confirmed this by blocking the activity of the 26S proteasome with MG132 and showed a steady-state increase in the level of these oncoproteins. When proteasome activity was inhibited in EGCG-treated HPV18 keratinocytes, we found that E6 and E7 protein degradation was reduced, albeit not to pre-treatment levels. Based on this observation, we hypothesise that EGCG promotes E6 and E7 degradation through a mechanism involving ubiquitination and proteasome-mediated degradation. As proteins earmarked for proteasomal degradation undergo post-translational modification prior to ubiquitination and proteolysis [34], we are currently investigating whether EGCG enhances E6 and E7 degradation by increasing the pool of poly-ubiquitinated E6 and E7 proteins.

One unexpected effect of EGCG treatment on the behaviour of HPV-infected keratinocytes was its ability to interfere with HPV18 lytic replication. Using the organotypic raft system as a model to induce the differentiation of HPV18-infected keratinocytes, we have shown that EGCG impairs lytic replication, as the expression of E4, a late protein involved in lytic replication, was abolished in EGCG-treated raft cultures generated from both HFK-HPV18 and VIN cl.11 cells. Although speculative, we hypothesise that this effect is also linked to the downregulation of E6 and E7, as both proteins are required to delay differentiation and to promote a replicative state necessary for genome amplification and virus maturation [32]. As the HPV life cycle is intimately linked to keratinocyte differentiation and EGCG has been shown to promote differentiation in normal keratinocytes, we examined the effects of EGCG treatment on the terminal differentiation of HPV18-positive keratinocytes. Interestingly, we found little, or no change in the expression of keratinocyte differentiation markers in EGCG-treated raft cultures, further strengthening our hypothesis that the inability of HR-HPV to undergo lytic replication is a consequence of E6 and E7 downregulation. Interestingly, in situ hybridization revealed that EGCG treatment did not result in an appreciable reduction in genome copy number in organotypic raft cultures, suggesting that the inability to undergo lytic replication was not due to loss of viral genomes.

To establish whether EGCG can be used to treat uVIN, we have established and utilised an authentic uVIN-derived cell line in our study. As far as we know, VIN cl.11 is the only VIN cell line available that harbours episomal forms of HPV18 and, when cultured in organotypic raft culture, can support lytic HPV replication. Although the cells harbours episomal forms of HPV18, we were not able to detect E6 and E7 expression at mRNA or protein level, precluding an assessment of EGCG’s ability to downregulate E6 and E7 in this cell line. We are currently unsure why we were unable to detect E6 and E7 expression in VIN cl.11, although one possible explanation is that their levels were below the limit of detection by immunoblotting. Nevertheless, when VIN cl.11 was cultured in organotypic raft culture, it formed dysplastic epithelial structures reminiscent of uVIN. As with HFK-HPV18, EGCG treatment inhibited the proliferation of VIN cl.11 raft culture and impaired its ability to support lytic HPV replication. Moreover, EGCG treatment repolarised the “expanded” ΔNp63-positive basal cell layer, giving rise to a single layer of basal cells juxtaposed to the basement membrane. Furthermore, we also showed that EGCG treatment resulted in re-expression of TSGs in VIN cl.11 raft cultures and speculate that like HFK-HPV18, EGCG reduces E6 and E7 expression in VIN cl.11, leading to re-expression of their target TSGs and cell growth inhibition.

## 4. Materials and Methods

### 4.1. Cell Lines

Information about the cell lines and their culture media are listed in Supplementary Table S1.

### 4.2. Establishment of a Primary uVIN-Derived Keratinocyte Cell Line

Tissue biopsies taken from a patient with histologically proven to have high-grade uVIN were explanted in E-medium as described [27]. Primary keratinocytes that grew out from the tissue explants were expanded on lethally irradiated 3T3-J2 feeder cells at clonal density (10^5^ cells/9 cm dish) in complete E-medium containing EGF. Individual colonies were selectively trypsinised using metal cloning cylinders and transferred to 6 cm Petri dishes containing lethally irradiated 3T3-J2 feeder cells. Individual clones were expanded prior to analysis. HPV genotyping of the clones was performed as described below. This study was approved by the local research ethics committee (Reference: 11/WM/0070).

### 4.3. HPV Genotyping with Luminex PCR

DNA was extracted from cell pellets using the DNAeasy kit (Qiagen, Manchester, UK) per manufacturer’s protocol. DNA was subjected to HPV genotyping using a Luminex-based PCR assay by the Scottish HPV Reference Laboratory [29].

### 4.4. Antibodies and Inhibitors

Information about inhibitors and antibodies used in this study are listed in Supplementary Table S2.

### 4.5. Cell Growth and Viability Assays

A431 and HeLa cells were seeded in triplicate into a 96-well plate at 3 × 10^3^ cells/well. For keratinocytes, 2 × 10^3^ cells were plated onto a monolayer of lethally irradiated 3T3 J2 feeder cells (1 × 10^4^ cells/well). Cells were cultured overnight (A431/HeLa) or for 48 h (keratinocytes) before treating with EGCG (Tocris, Abingdon, UK) at 0, 20, 40, 60, 80 and 100 μM. Cell proliferation was assessed 72 h later using the 5-bromo-2′-deoxyuridine (BrdU) ELISA assay kit (colorimetric immunoassay, Roche Diagnostics, Sussex, UK) as per the manufacturer’s protocol.

### 4.6. TUNEL Assay

The DeadEndTM Colorimetric TUNEL assay (Promega, Southampton, UK) was used to detect apoptotic cells. In brief, keratinocytes were seeded at a density of 5 × 10^4^ onto monolayer feeder cells (2.5 × 10^5^) grown on sterile 22 × 22 mm coverslips (Leica Biosystems, Milton Keynes, UK). Cells were left for 48 h, and feeder cells were removed. Cell medium was refreshed, and cells were treated with 0, 50 or 100 μM EGCG for 72 h or 2 with 5 μM Cisplatin for 24 h as a positive control. The presence of cellular apoptosis was detected per the manufacturer’s protocol.

### 4.7. Semi-Quantitative mRNA Analysis of E6/E7 Transcripts Using Real-Time PCR

HFK-HPV18 keratinocytes were grown to 60% confluency, and the feeder cells removed. Keratinocytes were then treated with 0, 50 and 100 μM EGCG for 3 or 6 days and then harvested. Total RNA was extracted from the cells using the RNeasy mini kit (Qiagen, Manchester, UK), and complementary DNA (cDNA) was synthesised through reverse transcription using QuantiTect reverse transcription kit (Qiagen, Manchester, UK), per manufacturer’s protocols. Relative quantification of HPV 18 E6/E7 was obtained using Fast Start PCR Master mix (Roche Diagnostics, Sussex, UK) and the following primers: forward primer (5′-AGAGGCCAGTGCCATTCGT-3′), reverse primer (5′-GTTTCTCTGCGTCGTTGGAGT-3′); and probe (5′-TCCTGTCGCTGGTTGCAGC-3′), purchased from Eurofins MWG operon and designed by Lindh et al. 2007^28^. HPV 18 E6/E7 transcripts were amplified by real-time PCR with ABI 7700 Sequence Detection System (Applied Biosystems/Thermofisher, Paisley, UK). PCR conditions were: initial enzyme activation step (50 °C/2 min), a denaturation step at (95 °C/10 min), followed by 40 cycles of denaturation (95 °C/15 s) and annealing/extension step (60 °C/1 min). Expression levels were normalised to the levels of endogenous beta-2 microglobulin gene in samples (Applied Biosystems/Thermofisher, Paisley, UK). Data were analysed using the relative 2ΔΔCT method using 7500 SDS software (Applied Biosystems/Thermofisher, Paisley, UK). All experiments were repeated twice more.

### 4.8. Western Blot Analysis

Cells were lysed in RIPA buffer, and 30 μg of total protein lysate was subjected to SDS-PAGE and transferred onto nitrocellulose membranes (VWR). The membranes were incubated with primary antibodies (see Supplementary Table S2) at 4 °C overnight followed by secondary anti-mouse or anti-rabbit antibodies. Protein bands were visualised with Fusion FX System (Vilber Lourmat) using SuperSignal West Dura Chemiluminescent Substrate (Thermo Scientific). Equal protein loading was verified using anti-β-actin antibody. The density of individual protein bands on the nitrocellulose membranes was quantified using the ImageJ 1.48v software; the value of each target protein was normalised to the respective density of the “housekeeping” protein (β-actin or GAPDH). All experiments were repeated twice more.

### 4.9. Immunocytochemistry and In Situ Hybridization (ISH)

Immunocytochemical staining was performed as previously described [14]. The antibodies used are listed in Supplementary Table S2. ISH was performed using a DNA probe specific for HPV16, 18, 31, 33 and 51 according to the manufacturer’s protocol (Bond™ Ready-to-Use ISH HPV Probe, Leica, UK).

### 4.10. Organotypic Raft Cultures

Organotypic raft cultures were prepared as previously described [36]. Briefly, 5 × 10^5^ keratinocytes were seeded onto a collagen lattice (3 mg/mL) (Becton Dickinson, Oxford, UK) containing 3T3-J2 fibroblasts (10^5^ cells/mL). Once confluent, the lattice was transferred to a stainless-steel grid and exposed at the air/liquid interface for 7 days. The appropriate concentration of EGCG was added to the medium, and the rafts cultured for an additional 7 days. Prior to formalin fixation, the rafts were incubated with 25 μg/mL 5-bromo-2′-deoxyuridine (BrdU) for 12 h; 4 μm sections were processed for H&E or immunohistochemical staining (Propath UK Ltd., Hereford, UK).

### 4.11. Statistical Analysis

Unpaired Student *t*-test was used to determine the level of significance for the difference in the proportion of apoptotic cells in drug-treated and untreated cells. The difference was considered significant if *p* < 0.05.

## 5. Conclusions

In conclusion, we have demonstrated that EGCG inhibits the growth of premalignant HPV18-positive keratinocytes by stimulating E6 and E7 viral oncoproteins’ turnover through the ubiquitin–proteasome pathway. Mechanistically, these effects appear to be due to re-expression of E6- and E7-targeted TSGs, which inhibit cell growth and induce apoptosis. Interestingly, EGCG treatment repolarizes the dysplastic basal cell layer in VIN cl.11 raft cultures, returning the premalignant epithelium to one that resembles normal epithelium in terms of its tissue architecture. Our findings support the use of topical EGCG treatment as a viable therapy for patients with recurrent uVIN and offer an alternative to surgical intervention. Our Phase II clinical trial (EPIVIN), which evaluated the use of topical EGCG ointment for the treatment with uVIN has revealed promising results. Future studies will utilize this material to validate our laboratory findings on the pre-and post-treatment tissue samples collected from patients who participated in the trial. Further work is currently being undertaken to evaluate the precise mechanism(s) by which EGCG promotes E6 and E7 protein ubiquitination and proteasome-mediated proteolysis.

## Data Availability

Data is contained within the article or Supplementary Materials.

## Supplementary Materials

The following are available online at https://www.mdpi.com/article/10.3390/pathogens10040459/s1, Figure S1: Representative karyotypes from two major clones identified in cultured VIN cl.11 cells. Chromosome alignment of G-banded chromosomes taken from two additional major clones (A) clone 1, and (B), clone 3 found in early-passage cultures of VIN cl.11. This analysis confirmed the near-tetraploid nature of chromosomes and the absence of a Y chromosome. Figure S2: The morphology of VIN cl.11 in monolayer culture. The morphology of (i) normal vulvar keratinocytes, (ii) VIN cl.11 and (iii) HFK-HPV18 in monolayer cultures. Keratinocytes were seeded at clonal density on irradiated 3T3-J2 fibroblasts. After 7 days, the feeder cells were removed, and a phase-contrast image taken using a Nikon Eclipse E600 microscope at ×200 magnification. Table S1: Cell lines and culture media. Table S2: Antibodies and Chemicals used in this study.
