# Supplementary material for: The Major Constituent of Green Tea, Epigallocatechin-3-Gallate (EGCG), Inhibits the Growth of HPV18-Infected Keratinocytes by Stimulating Proteasomal Turnover of the E6 and E7 Oncoproteins
Source: Pathogens. 2021 Apr 11;10(4):459. doi: 10.3390/pathogens10040459 (PMC8069595; doi:10.3390/pathogens10040459)
Supplement: Supplementary file 1 [file pathogens-10-00459-s001.pdf]

pathogens

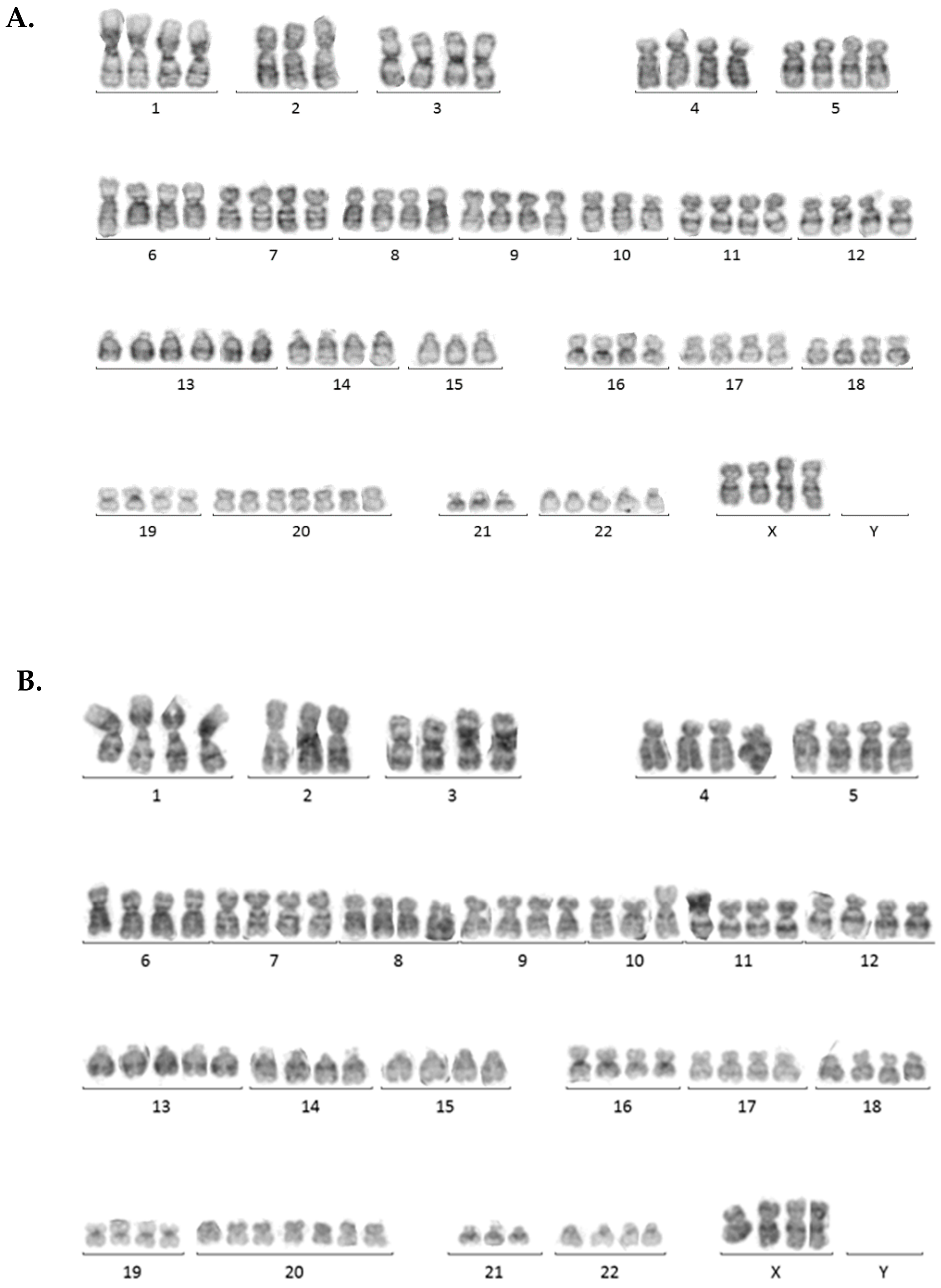

Figure S1. Representative karyotypes from two additional major clones identified in primary cultures of VIN cl.11. Chromosome alignment of G-banded chromosomes taken from two additional major clones (A) clone 1, and (B), clone 3 found in early passage cultures of VIN cl.11. This analysis confirmed the near-tetraploid nature of chromosomes and the absence of a Y chromosome.

i **Normal Keratinocytes**

ii **VIN cl.11**

iii **HFK-HPV18**

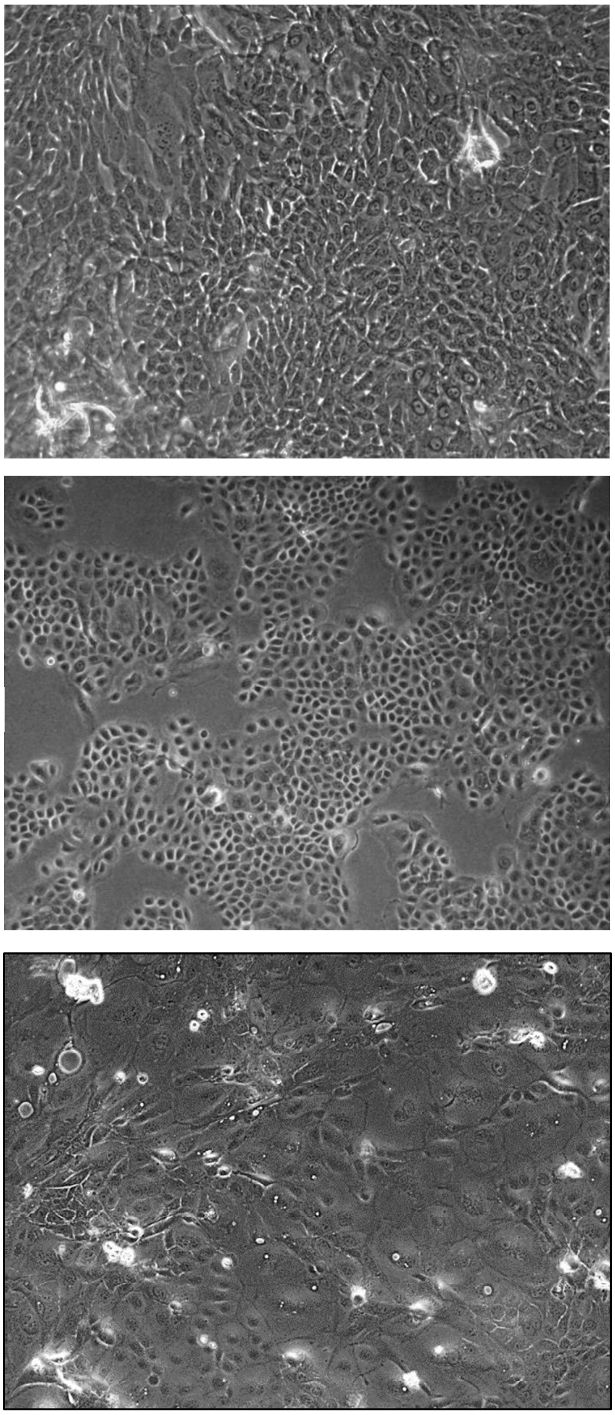

Figure S2. The morphology of VIN cl.11 in monolayer culture. The morphology of (i) normal vulvar keratinocytes, (ii) VIN cl.11 and (iii) HFK-HPV18 in monolayer cultures. Keratinocytes were seeded at clonal density on irradiated 3T3-J2 fibroblasts. After seven days, the feeder cells were removed and a phase contrast image taken using a Nikon Eclipse E600 microscope at ×200 magnification.

Table S1. Cell lines and culture media.

<table>
<tr><th>Cell line</th><th>Source</th><th>Origin</th><th>Tissue culture media and supplements</th></tr>
<tr><td>A431</td><td>E Odintsova, University of Birmingham</td><td>Derived from vulval epidermal carcinoma of a 85-year old women</td><td>Dulbecco's Modified Eagle's Medium (DMEM) (Gibco, UK) supplemented with 10% v/v fetal bovine serum (FBS), 100U/ml penicillin, 100mg/l streptomycin and 4mM L-glutamine (Gibco, UK)</td></tr>
<tr><td>HeLa</td><td rowspan="3">S Roberts University of Birmingham</td><td>Derived from cervical carcinoma of a 31-year old female; contains integrated form of HPV 18 genomes</td><td>DMEM (Gibco, UK) supplemented with 10% v/v fetal bovine serum (FBS), 100U/ml penicillin, 100mg/l streptomycin and 4mM L-glutamine (Gibco, UK).</td></tr>
<tr><td>HFK-HPV18</td><td>Derived from infant foreskin keratinocytes and transfected with episomal form of HPV 18</td><td>E-media: DMEM 60% v/v (Gibco, UK), Ham's F12 32% v/v (Gibco, UK), PenStrep 10,000 U/mL 2% v/v (Gibco, UK), hydrocortisone 0.1% v/v (Sigma, UK), HyClone Defined FBS 10% v/v (Fisher Scientific, UK), mouse epidermal growth factor (EGF) 5ng/ml (BD Biosciences, UK), L-glutamine 2mM (Gibco, UK), cholera toxin A 0.1% v/v (Sigma Aldrich, UK), insulin 0.2% v/v (Sigma Aldrich, UK), transferrin 0.2% v/v (Sigma Alrich, UK), tri-iodo–lL-thyronine T3 4 nM (Sigma Aldrich, UK) and adenine 36 μM (Sigma Aldrich, UK)</td></tr>
<tr><td>3T3-J2</td><td>Mouse embryonic fibroblast cell line. Used after γ-irradiation as feeder cells for keratinocytes.</td><td>DMEM, HEPES modified, (Sigma-Aldrich, UK) supplemented with 10% v/v new born calf serum, 4mM L-glutamine and 100U/ml penicillin and 100mg/l streptomycin</td></tr>
<tr><td>VIN cl.11</td><td>Generated as part of this study</td><td>Primary premalignant keratinocyte clone isolated from a uVIN biopsy</td><td>1:1 RPMI 1640 media and Ham's F12 nutrient mixture media containing 5% v/v foetal calf serum (FCS), 0.4μg/ml hydrocortisone, 10ng/ml EGF, 100U/ml penicillin, 100mg/l streptomycin</td></tr>
</table>

Table S2. Antibodies and Chemicals used in this study.

Antibody	1° or 2°	Manufacturer	Catalogue code	Species	Dilutions
β-actin	1°	Sigma-Aldrich, UK	A5316	Mouse	WB 1:1000
ΔNp63	1°	Santa-Cruz	sc-8431	Mouse	IHC 1:200
HPV18 E4	1°	Dr S Roberts University of Birmingham [14]	n/a	Mouse	IHC 1:5
HPV18 E6	1°	Santa-Cruz	sc-365089	Mouse	WB 1:100
HPV18 E7	1°	Abcam	ab100953	Mouse	WB 1:1000
Involucrin	1°	Sigma	SY5	Mouse	WB 1:200<br>IHC 1:100
K1/K10	1°	Sigma-Aldrich, UK	8.60	Mouse	IHC 1:100
Ki67	1°	Dako	MIB-1	Mouse	IHC 1:100
MCM7	1°	Sigma-Aldrich, UK	17931	Mouse	WB 1:2000<br>IHC 1:200
$p16^{INK4a}$	1°	Abcam	ab7962	Mouse	WB 1:200<br>IHC 1:50
$p21^{WAF1}$	1°	Santa-Cruz	sc-397	Rabbit	WB 1:100<br>IHC 1:100
p53	1°	Dr RJ Grand University of Birmingham	n/a	Mouse	WB 1:100<br>IHC 1:100
pRb	1°	Cell Signalling	9309S	Mouse	WB 1:1000<br>IHC 1:100
Anti-Mouse-HRP	2°	Dako	P0447	Goat	WB 1:1000
Anti-Rabbit-HRP	2°	Dako	P0448	Goat	WB 1:1000
Anti-Mouse AlexaFluor488	2°	Invitrogen	A32723	Goat	ICC 1:1000
Anti-Rabbit AlexaFluor488	2°	Invitrogen	A32731	Goat	ICC 1:1000

Chemical	Manufacturer	Catalogue code	Diluent
EGCG	Tocris, UK	4524	H20
MG132	Caymen Chemical, UK	10012628	DMSO
Cisplatin	Tocris, UK	2251	H20
Cycloheximide	Sigma-Aldrich, UK	C4859	DMSO
